# The Retail Food Environment: Time for a Change

**DOI:** 10.3390/ijerph17238846

**Published:** 2020-11-28

**Authors:** Alyssa Moran, Christina Roberto

**Affiliations:** 1 Department of Health Policy and Management, Johns Hopkins Bloomberg School of Public Health, Baltimore, MD 21205, USA; 2 Department of Health Policy and Medical Ethics, Perelman School of Medicine at the University of Pennsylvania, Philadelphia, PA 19104, USA; croberto@pennmedicine.penn.edu

## 1. Introduction

Food retailers, manufacturers, and distributors exert powerful influence on our food choices through decisions about stocking, pricing, marketing, and promotional practices [1]. Such practices often encourage selection and consumption of foods and beverages that are nutritionally poor [2] and may exacerbate the global burden of obesity and diet-related chronic diseases [3]. Interventions that alter the retail food environment to support healthy eating are urgently needed. In the United States, this is particularly pressing in communities of color, rural areas, and low-income neighborhoods, where unhealthy food marketing is highly prevalent [4]. In this Special Issue, we summarize the current evidence on the links between the food retail environment and health behaviors and outcomes and identify future research priorities.

This Special Issue was borne out of a convening held in January 2020, sponsored by Center for Science in the Public Interest, The Food Trust, the Johns Hopkins Bloomberg School of Public Health, and Healthy Eating Research (HER), a national program of the Robert Wood Johnson Foundation. Recognizing that sustainable interventions require cross-sector and multidisciplinary collaboration, meeting attendees included food industry representatives, non-profit organizations, and researchers spanning a wide range of disciplines relevant to healthy food retail. The objective of the convening was to develop a national healthy retail research agenda, which aimed to identify effective retail interventions to support nutritious food choices, with an intentional focus on reducing disparities in food marketing and access. This Special Issue includes research commissioned for or resulting from the meeting, including one paper describing the research agenda, one communication, three empirical papers, and four reviews. Papers are organized into four sections: (1) the retail food environment and industry practices; (2) consumer food shopping patterns; (3) effectiveness of retail interventions to support healthy eating; and (4) the future of food retail research.

## 2. The Retail Food Environment and Industry Practices

Relationships between the retail food environment and consumer food and beverage choices are complex. Prior conceptual frameworks do not fully capture factors influencing where retailers locate, which products they choose to sell, how products are marketed or merchandised in the store, and how these decisions interact with other factors to influence consumer selection. To fill this gap, Hecht and colleagues [5] review industry and academic literature to catalogue trade promotion practices used by manufacturers and distributors to influence retail food marketing strategies and to examine the effects of these practices on consumer purchases. The authors identify four categories of trade promotion practices—category management, cooperative advertising, price discounts, and slotting allowances—which influence pricing, placement, and promotion of products by retailers.

The categories of promotional practices catalogued by Hecht and colleagues are incorporated into two new conceptual frameworks describing the influence of the retail food environment on consumer behaviors. The Retail Food Environment and Customer Interaction Model, put forth by Winkler and colleagues [6], conceptualizes the retail food environment as a complex dynamic system, including reciprocal relationships between characteristics of retailers (sources, actors, business models) and customers (individual, interpersonal, and household factors), as well as macro-level contexts, such as policies and economic systems, that influence these relationships. The authors contend that interactions between these factors can influence important population outcomes, which include health, but also food security, environmental sustainability, business sustainability, and food sovereignty, equity, and justice. A second conceptual framework, developed by Khandpur and colleagues [7], takes a closer look at the online food retail environment. This framework, which is nested in the socioecological model, identifies both consumer- and retailer-level influences along the online path-to-purchase, including consumer demographic characteristics, preferences, and past behaviors, as well as equity and transparency of retailer policies and practices, which interact to influence decision-making. The framework draws from multiple disciplines, emphasizing the dynamic nature of personalized marketing by retailers and customizable website content, which separates food marketing in the online setting from that within the brick-and-mortar store.

## 3. Consumer Food Shopping Patterns

This Special Issue includes research designed to better understand consumer food shopping patterns, particularly across understudied populations. Original research by Lacko and colleagues [8] updates and extends prior work examining trends in grocery sales since 2012. The authors document the top sources of calories across different retail store types, break this down by urban or rural household residence and household income, and then examine the interaction between household income and urbanicity. Their research reveals differences in nutritional quality of packaged foods purchased across store types, with purchases of lower nutritional quality made at dollar stores and convenience stores compared to club stores and supermarkets. They find that rural shoppers purchase more calories per person per day from mass merchandisers and dollar stores compared to urban households. The paper reports little influence of urbanicity or household income on food purchases within store type.

The new empirical data presented by Lacko and colleagues is complemented by a systematic review by Singleton and colleagues [9] that was designed to summarize studies examining the influence of intersectionality—interactions between race/ethnicity, socioeconomic status, and geographic location—on consumer food purchasing. The paper reviews literature describing differences in food purchases within attributes like socioeconomic status but reveals a dearth of studies examining how sociodemographic factors interact. In their paper, they propose areas where future work is needed to address these major gaps to better inform efforts to implement healthy food retail strategies in underserved, low-resourced, and marginalized communities.

## 4. Effectiveness of Retail Interventions to Support Healthy Eating

This Special Issue includes two systematic reviews examining effects of interventions to support healthy eating. Karpyn and colleagues [10] review 64 in-store marketing studies published between 2010 and 2019 intending to promote nutritious food choices. The authors find that in-store interventions are often multi-component, including changes to in-store promotions (e.g., signage or shelf labels), pricing (e.g., discounts or subsidies), placement (e.g., product prominence or display); and/or product (e.g., availability within the store). Most interventions are associated with at least one positive outcome related to dietary behaviors; however, few studies have used strong experimental or quasi-experimental designs or objective outcome measures.

A systematic review written by Moran and colleagues [11] complements the work of Karpyn and colleagues by examining the effects of government policy interventions. The paper reviews 147 academic papers describing associations between governmental policies intended to promote healthy food and beverage choices in supermarkets and a wide range of individual, retailer, and community health outcomes. Findings show positive associations between three policies and dietary behaviors: financial incentives for fruits and vegetables provided to low-income households, revisions to the USDA Special Supplemental Nutrition Program for Women, Infants, and Children food package, and sugary drink excise taxes. Two policies—increases in Supplemental Nutrition Assistance Program (SNAP) benefits and incentives for supermarkets to open in underserved areas—show limited effects on dietary intake and food purchasing, but may improve food security. The paper identifies significant gaps in knowledge about children, rural populations, and people living in the Midwestern and Southern United States.

## 5. The Future of Food Retail Research

The final full-length paper in the series by Hecht and colleagues [12] describes the scope of the healthy retail research agenda, which includes ten priority areas designed to understand current food retail environments and their influence on consumer behavior and effectiveness of interventions to create healthier retail environments. The paper also details the agenda-setting process, and recommendations from expert stakeholders on healthy retail research approaches, data sources, and areas of future research.

The series is then completed with a timely communication from Leone and colleagues [13] that reflects on the impact COVID-19 has had on food systems and environments in the U.S. Using Winkler and colleagues’ Retail Food Environment and Customer Interaction Model, the authors describe how COVID-19 has impacted policies, retailers, and customer experiences and dietary intake. The commentary also discusses how the COVID-19 pandemic has further exacerbated long-standing inequities in food insecurity, food access, and health across race, ethnicity, class and geography. The authors then identify a series of research priorities that are needed to create a more just and equitable retail food environment and improve the country’s emergency preparedness.

## 6. Conclusions

Taken together, these papers advance our understanding of the complex relationships between the retail food environment, dietary behaviors, and the public’s health. This Special Issue highlights substantial gaps in our knowledge of consumer food purchasing patterns and impacts of healthy retail interventions by race, ethnicity, class and geography—research that is critical for addressing health inequities. The culminating healthy retail research agenda synthesizes this work and details a path forward, describing partnerships, data sources, research methods, and priority questions for advancing the field.

## References

[1] Glanz K., Bader M.D.M., Iyer S. (2012). Retail grocery store marketing strategies and obesity. Am. J. Prev. Med..

[2] Rivlin G. (2016). Rigged: Supermarket Shelves for Sale.

[3] Gortmaker S.L., Swinburn B., Levy D., Carter R., Mabry P.L., Finegood D., Huang T., Marsh T., Moodie M.L. (2011). Changing the future of obesity: Science, policy, and action. Lancet.

[4] Berkeley Media Studies Group (2019). The 4 PS of Marketing: Selling Junk Food to Communities of Color.

[5] Hecht A.A., Perez C.L., Polacsek M., Thorndike A.N., Franckle R.L., Moran A.J. (2020). Influence of food and beverage companies on retailer marketing strategies and consumer behavior. Int. J. Environ. Res. Public Health.

[6] Winkler M.R., Zenk S.N., Baquero B., Anderson Steeves E., Fleischhacker S.E., Gittelsohn J., Leone L.A., Racine E.F. (2020). A model depicting the retail food environment and customer interactions: Components, outcomes, and future directions. Int. J. Environ. Res. Public Health.

[7] Khandpur N., Zatz L.Y., Bleich S.N., Taillie L.S., Orr J.A., Rimm E.B., Moran A.J. (2020). Supermarkets in cyberspace: A conceptual framework to capture the influence of online food retail environments on consumer behavior. Int. J. Environ. Res. Public Health.

[8] Lacko A., Ng S.W., Popkin B. (2020). Urban vs. rural socioeconomic differences in the nutritional quality of household packaged food purchases by store type. Int. J. Environ. Res. Public Health.

[9] Singleton C.R., Winkler M., Houghtaling B., Adeyemi O.S., Roehll A.M., Pionke J.J., Anderson Steeves E. (2020). Understanding the intersection of race/ethnicity, socioeconomic status, and geographic location: A scoping review of U.S. consumer food purchasing. Int. J. Environ. Res. Public Health.

[10] Karpyn A., McCallops K., Wolgast H., Glanz K. (2020). Improving consumption and purchases of healthier foods in retail environments: A systematic review. Int. J. Environ. Res. Public Health.

[11] Moran A.J., Gu Y., Clynes S., Goheer A., Roberto C.A., Palmer A. (2020). Associations between governmental policies to improve the nutritional quality of supermarket purchases and individual, retailer, and community health outcomes: An integrative review. Int. J. Environ. Res. Public Health.

[12] Hecht A.A., Lott M.M., Arm K., Story M.T., Snyder E., Wootan M.G., Moran A.J. (2020). Developing a national research agenda to support healthy food retail. Int. J. Environ. Res. Public Health.

[13] Leone L.A., Fleischhacker S., Anderson-Steeves B., Harper K., Winkler M., Racine E.F., Baquero B., Gittelsohn J. (2020). Healthy food retail during the COVID-19 pandemic: Challenges and future directions. Int. J. Environ. Res. Public Health.

